# What is the current significance of low birthweight pigs on commercial farms in Northern Ireland in terms of impaired growth and mortality?

**DOI:** 10.1093/tas/txaa147

**Published:** 2020-07-31

**Authors:** Samuel J Hawe, Nigel Scollan, Alan Gordon, Elizabeth Magowan

**Affiliations:** 1 Agri-Food and Biosciences Institute, Livestock production Sciences Unit, Hillsborough, UK; 2 Queens University Belfast, Institute for Global Food Security, Belfast, UK

**Keywords:** large litters, low birthweight, lifetime growth, mortality, weaning

## Abstract

There is little modern data addressing the differential lifetime growth of commercially reared low and average birthweight pigs born into large litters (>14 piglets). As such, the main aim of this study was to quantify the lifetime growth and mortality rate of low and average birthweight pigs on commercial farms in Northern Ireland. It was also aimed to analyze the level, stage and cause of mortality within each birthweight category. A total of 328 low birthweight (low BW; <1 kg) and 292 average birthweight (Av BW; 1.3 to 1.7 kg) pigs were individually identified across four commercial farms and one research farm. Animal growth and mortality were monitored on an individual basis from birth until slaughter age. Av BW pigs were heavier than low BW pigs throughout the trial (*P* < 0.001), with a weight advantage of 1.16 kg at weaning increasing to over 9 kg at slaughter age. Av BW pigs recorded a superior average daily gain (ADG) to low BW pigs throughout the trial (*P* < 0.05), with the greatest difference recorded immediately postweaning between weeks 4 and 8 and weeks 8 and 12 when a 77 and 85 g/d difference was recorded, respectively. AV BW pigs which were cross-fostered were significantly lighter than those remaining with their birth mother at weaning (0.9 kg), week 8 (1.7 kg), and week 12 (3.1 kg) (*P* < 0.05, respectively). The variance of weight was significantly greater for the AV BW pig population than the low BW pig population at week 4 (*P* < 0.001) and 8 (*P* < 0.05). Preweaning mortality of low BW pigs was over three times greater than that of Av BW pigs (21% vs. 6%; *P* < 0.001), with low BW deaths occurring earlier (9.2 d vs. 15.4 d; *P* < 0.001) and at a lighter weight (1.2 vs. 2.4 kg; *P* < 0.001) than Av BW pigs. There was a clear association between birthweight and cause of preweaning death (*P* < 0.05), with starvation (49%) and overlying (28%) accounting for the majority of low BW mortalities. Birthweight had no effect on rate, age, or weight of postweaning mortalities (*P* > 0.05). The alimentary tract (27%) and respiratory tract (27%) were the most commonly implicated body systems following postmortem examination of postweaning deaths. In conclusion, this study quantified the inferior weight, growth rate, and mortality of low BW pigs, identifying the lactation and immediate postweaning periods as having the greatest potential in reducing this birthweight associated growth differential.

## INTRODUCTION

In recent years, the incorporation of prolific genetics, combined with improved management systems, has resulted in significant increases in pig litter size (Bruns et al., 2018). In Northern Ireland (NI), this has resulted in an increase of 3.5 pigs born alive per litter as well as an increase of 5.3 pigs weaned per sow per year over the last 10 years, resulting in sows in NI weaning an average of 29.8 pigs per year in 2019 (Donnelly, 2019). Consequently, there has been an increase in the number of low birthweight and potentially “non-viable” piglets born, which is in agreement with the findings from other pig industries (Varona et al., 2007; Quiniou et al., 2002). The increase in low birthweight piglets at birth is largely attributed to intrauterine growth retardation (IUGR), whereby the uterine blood flow in modern commercial sows is not sufficient to provide adequate nutrients to the increased number of fetuses (Antonides et al., 2015).

Arguably, the major issue with low birthweight piglets is their elevated level of preweaning mortality. Marchant et al. (2000) showed pigs with birthweights of 1.1 kg or less can display levels of preweaning mortality up to 28%. When compared with the average preweaning mortality of 12.7% recorded within the Northern Ireland pig industry (Donnelly, 2018), this highlights the obvious financial and welfare implications of these low birthweight pigs. Low birthweight animals also exhibit reduced weaning weights and poor lifetime performance (Fix et al., 2010). Indeed, Williams (2003) showed how the divergence in weight at weaning increased throughout the growing period and Beaulieu et al. (2010) quantified that pigs with a birthweight of 1.20 kg or less required an additional 10 d on average to reach slaughter weight. Rehfeldt and Kuhn (2006) concluded that the impaired muscle fiber network evident in low birthweight pigs resulted in a reduced lean growth potential. Excess energy is therefore diverted to lipid accretion, which impairs feed efficiency and carcase quality. Hence, low birthweight pigs are a chronic and increasing problem for performance and profitability in commercial farms.

It is accepted that the majority of piglets are born within a weight range of 1.3 and 1.7 kg (Quiniou et al., 2002). However, much of the existing literature comparing the performance of low birthweight pigs to heavier counterparts has been conducted on litter sizes of 11 pigs or less and a birthweight of over 1 kg, which is not reflective of current commercial practice (Douglas et al., 2014). Although some recent studies have analyzed the performance and mortality of compromised pigs reared in large litters, this was not balanced with a comparison to heavier littermates (Ward et al., 2020; Feldpausch et al., 2019). These studies were also conducted under controlled conditions, where animal responses may differ from those recorded in the field (Magowan et al., 2007). With an increase in litter size projected to continue, there is a need to accurately quantify the impact of low birthweight pigs on commercial production to identify where future research efforts should focus.

This study was undertaken to quantify the individual lifetime growth performance of low birthweight pigs and compare this to ‘average’ birthweight littermates on commercial farms in Northern Ireland. It was also designed to establish the scale, stage and cause of mortality for each birthweight category, as well as analyze growth variation between farms. It was hypothesized that low birthweight pigs would express a greater level of mortality and impaired lifetime growth performance when compared with average birthweight animals. Furthermore, differences in weight were expected to become more pronounced as animals progressed through the production cycle.

## MATERIALS AND METHODS

This study was conducted on four high-performance commercial units and one research farm within the Northern Ireland pig industry. All farms were quality assured and complied fully with The Welfare of Farmed Animals Regulations (Northern Ireland Assembly, 2012). To maintain confidentiality within this study, each farm was randomly assigned a unique identification number (Farms 1 to 5). As this experiment was designed to monitor and compare the health and performance of low birthweight (low BW) and average birthweight (Av BW) pigs in the commercial setting, no specific treatments were imposed. Indeed all animals were reared within the management and production regimes employed on each farm.

### Animal Selection

This study employed 328 low BW and 292 Av BW piglets from birth to slaughter. low BW animals weighed an average of 0.92 ± 0.01 kg at birth and ranged from 0.5 to 1 kg, whereas Av BW animals recorded an average weight of 1.51 ± 0.01 kg at birth and ranged from 1.3 to 1.7 kg. Animals on each farm were selected from a single farrowing batch. Following the completion of farrowing, piglets were selected from as many sows as possible to minimize any sibling effect. The comprehensive demographics of the animals selected on each farm are outlined in Supplementary Table S1. So far as possible, an equal number of boars and gilts were selected per birthweight category on each farm, such that sex did not differ significantly between the two birthweight categories. On each farm, low BW and Av BW piglets were also evenly spread across a range of sow parities, such that parity of origin was balanced for low BW and AV BW pig populations on each farm.

### Animal Management

The animal usage, genetic profile, housing conditions, and basic management practices employed on each farm are outlined in Supplementary Table S1. The dietary regime employed on each farm is shown in Supplementary Table S2. All diets employed on each farm met the energy and lysine requirements of animals for each stage of production outlined by Whittemore et al. (2003).

### Measurements and Data Collection

Each animal was individually weighed at birth using UWE HS-15K hanging scales (County Scales Limited, Nottingham, UK) and again on the day before weaning. Animals were further weighed individually at 8, 12, 17, and 22 wk of age using an LS-521 Livestock Weigher (Brecknell Scales, West Midlands, UK). Management strategies and housing dimensions were recorded for each stage of production on all farms participating in the trial. The specification of all diets offered was also recorded along with the time at which diets changed.

### Mortalities and Postmortem Examination

All animal deaths had a ‘death date’ and ‘death weight’ recorded throughout both the pre- and post-weaning periods. The cause of preweaning death was recorded by farmers using the template outlined in Supplementary Appendix 1, and hence these results should be viewed cautiously. Postweaning mortalities were subject to postmortem analysis (AFBI Veterinary Services Division). Before arrival, each carcass for postmortem analysis was labeled with the corresponding farm, farm vet responsible for the farm, project number and name, animal history, and contact number. Following postmortem analysis, causes of death were grouped according to the body system in which they occurred. This allowed the generation of a more uniform dataset which was suitable for statistical analysis.

### Statistical Analysis

Continuous variables were analyzed using a linear mixed model methodology, while binary variables were analyzed as a generalized linear mixed model (binomial distribution, logit link function). The random and fixed models were the same in both cases. The main aim of this study was to compare the performance of low BW and Av BW pigs. Therefore, for analysis of liveweight, growth rate and mortality, animal birthweight, birth mother parity, number of piglets born alive in each litter, number of stillborn (SB) piglets in each litter, total litter size and fostering were fitted as fixed effects. The first-order interaction between birthweight and fostering was also analyzed. Whilst the experimental unit was the individual pig, nested effects were accounted for by fitting farm and birth mother as random effects. A contingency table permutation test determined if any trends or differences existed in the cause of either pre- or postweaning deaths across both birthweight categories. A secondary aim of this study was to analyze variation between farms. Therefore, farm and birthweight were fitted as fixed effects for analysis comparing liveweight, variance in liveweight and average daily gain (ADG) between farms, with birth mother fitted as a random effect. The first-order interaction between farm and birthweight was also considered when comparing liveweight between farms. The variance of liveweight within each birthweight category was calculated as follows:

$$\begin{array}{l} S^{2}=\;\left[\sum {\left(x_{i}-\bar{x}\right)}^{2}\right]/n1 \end{array}$$

where *S*^2^ is sample variance, *x*_i_ is the value of one observation, *x̄* is the mean value of all observations, and *n* is the number of observations.

A two-sample *t*-test was employed to establish if there was a significant difference between the variance of weights of low BW and Av BW pigs at each weighing. A Levene’s test (Levene, 1960) was used to establish if the homogeneity of variance in weights of low BW and Av BW pigs differed between each of the farms under trial at each weighing. Weight was modeled against time using an exponential curve for 486 animals (235 low BW, 251 Av BW) on an individual basis. In each case, the model parameters were saved and then analyzed using a linear mixed model, with birthweight set as a fixed effect and farm included as a random effect. Pearson’s correlation coefficient was used to estimate the correlation between the weight recorded for low BW and Av BW pigs at different ages. All statistical analyzes were carried out using GenStat 16^th^ edition (Lawes Agricultural Trust, Rothamsted Experimental Station). Significance was defined as *P* < 0.05, with tendencies defined as *P* < 0.1.

## RESULTS

### Animal Growth Performance

**Figure 1.**
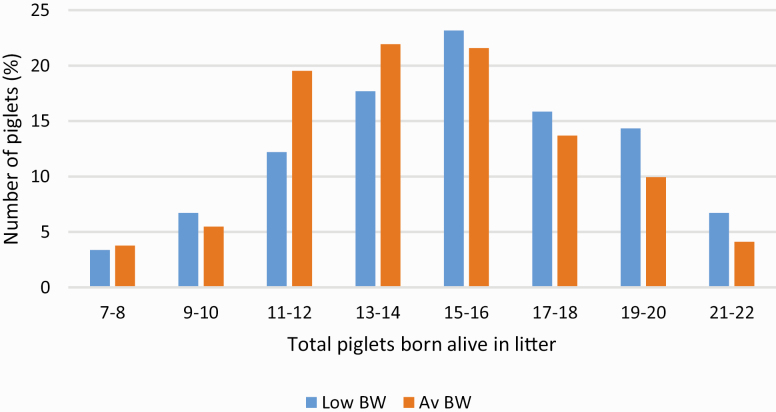
Number of piglets born alive in litter of origin for low birthweight and average birthweight pigs.

**Figure 2.**
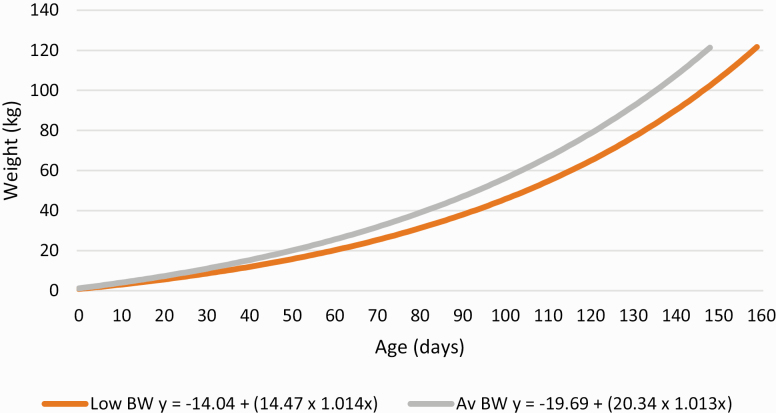
Model describing the time required for low and average birthweight pigs to achieve a slaughter weight of 120 kg, where *x* = age (d) and *y* = liveweight (kg).

The growth performance of low BW and Av BW pigs is reported in Table 1. Animal liveweight and ADG was not significantly affected by birth mother parity or the number of SB piglets in the litter. Animal liveweight was also not affected by total litter size. There was no significant interaction between birthweight and the average number of piglets born alive per litter or total litter size for any parameters reported in Table 1 (*P* > 0.05). The average number of piglets born alive per litter during the study was 15 ± 0.13 and ranged from 7 to 22. The litter of origin for low BW piglets recorded a significantly greater number of piglets born alive on average (15.3 vs. 14.6, *P* < 0.05) and tended to record a greater total litter size on average (16.4 vs. 15.7, *P* < 0.1) compared with Av BW piglets. Figure 1 shows that a greater percentage of Av BW piglets were sourced from litters recording 7 to 14 piglets born alive compared with low BW pigs (51% vs. 40%). However, a greater proportion of low BW piglets were sourced from litters recording 15 to 22 piglets born alive than for Av BW pigs (60% vs. 49%). As hypothesized, Av BW pigs were significantly heavier than low BW pigs throughout the trial (*P* < 0.001). This weight advantage of Av BW pigs increased from 0.59 kg at birth to 1.16 kg at weaning. Over the growing and finishing phases, this weight differential further increased to 3.4 kg at week 8, 5.7 kg at week 12, 7.5 kg at week 17, and 9.1 kg by week 22. This was driven by a greater ADG for Av BW pigs during the intervals between each weighing throughout the trial (*P* < 0.05). The greatest difference in ADG was recorded between weeks 4 and 8 as well as between weeks 8 and 12, when Av BW animals expressed 77 and 85 g/d superior growth rate, respectively. The time required for low BW and Av BW piglets to reach a slaughter weight of 120 kg is predicted by the model reported in Figure 2. This figure shows low BW pigs require an estimated additional 11 d to reach a market weight of 120 kg in comparison to Av BW counterparts.

**Table 1.** Effect of birthweight, sow parity, litter size, and fostering on pig liveweight and ADG from birth to 22 weeks of age

				*P*-value						
	Low BW	Av BW	SEM	Birthweight	BM parity	BA in litter	SB in litter	Total litter size	Fostered	Birthweight × fostered
Animal weight, kg										
Birth	0.92	1.51	0.008	<0.001	0.105	0.049	0.467	0.051	0.231	0.866
Week 4	6.76	7.92	0.149	<0.001	0.643	0.201	0.481	0.381	0.01	0.032
Week 8	15.7	19.1	0.31	<0.001	0.578	0.774	0.753	0.867	0.036	0.025
Week 12	31.6	37.3	0.55	<0.001	0.848	0.897	0.990	0.907	0.022	0.041
Week 17	58.2	65.7	0.91	<0.001	0.233	0.646	0.311	0.438	0.266	0.060
Week 22	91.7	100.8	1.19	<0.001	0.527	0.086	0.478	0.071	0.717	0.267
Average daily gain, g/d										
Birth–week 4	204	230	5.3	<0.001	0.659	0.185	0.497	0.296	0.018	0.025
Week 4–week 8	321	398	8.4	<0.001	0.508	0.725	0.899	0.773	0.237	0.156
Week 8–week 12	564	650	12.5	<0.001	0.857	0.535	0.692	0.651	0.043	0.185
Week 12–week 17	757	811	15.2	<0.001	0.068	0.377	0.191	0.210	0.469	0.233
Week 17–week 22	944	991	18.0	0.009	0.907	0.021	0.849	0.037	0.851	0.570
Birth–week 22	589	645	7.7	<0.001	0.53	0.084	0.476	0.066	0.735	0.265
Week 4–week 22	674	737	9.0	<0.001	0.514	0.098	0.355	0.066	0.977	0.386

Low BW, low birthweight pigs, <1 kg; Av BW, average birthweight pigs, 1.3 to 1.7 kg; BM parity, parity of birth mother; BA in litter, number of piglets born alive per litter; SB in litter, number of still born animals per litter.

There was no significant difference in the weight of low BW animals which were fostered and low BW animals which remained with their birth mother at week 4 (6.6 kg vs. 6.8 kg; *P* > 0.05), week 8 (15.6 kg vs. 15.8 kg; *P* > 0.05), and week 12 (31.2 kg vs. 32 kg; *P* > 0.05). However, Av BW animals which were fostered were significantly lighter than those remaining with their birth mother at week 4 (7.5 kg vs. 8.4 kg; *P* < 0.05), week 8 (18.2 kg vs. 19.9 kg; *P* < 0.05), and week 12 (35.6 kg vs. 38.7 kg; *P* < 0.05). The majority of cross-fostered animals were also of a low BW (57% low BW vs. 43% Av BW; *P* < 0.05). There was a significant correlation between birthweight, weaning weight and weight at slaughter age for both low BW and Av BW pigs (Table 2). All correlations were highly significant (*P* < 0.001) apart from those between birthweight and the 8, 12, 17, and 22 weeks weight of Av BW pigs (*P* < 0.05). In general, the correlation between birthweight and subsequent weights was stronger for low BW animals than Av BW pigs. However, the strength of the correlation between weaning weight and subsequent weights was similar for both birthweight categories.

**Table 2.** Correlation between pig liveweight at various ages for low birthweight and average birthweight animals

Age	Birth weight	4 week weight	8 week weight	12 week weight	17 week weight	22 week weight
Birth weight	—	0.22***	0.15*	0.14*	0.15*	0.17**
4 weeks weight	0.40***	—	0.56***	0.50***	0.43***	0.37***
8 weeks weight	0.32***	0.53***	—	0.84 ***	0.67***	0.56***
12 weeks weight	0.32***	0.49***	0.86***	—	0.83***	0.70***
17 weeks weight	0.33***	0.48***	0.72***	0.80***	—	0.85***
22 weeks weight	0.23***	0.50***	0.58***	0.67***	0.84***	—

—, Values below the diagonal report correlations between the weights of low BW pigs, whereas values above the diagonal report correlations between the weights of Av BW pigs.

*** *P* < 0.001; ** *P* < 0.01.; * *P* < 0.05.

### Impact of Birthweight, Age, and Farm on Weight Variation

The variance of animal weights was significantly lower for low BW pigs than for Av BW pigs at week 4 (*P* < 0.001) and week 8 (*P* < 0.05) (Table 3). However, there was no significant difference in the variance of weights of low BW and Av BW pigs at week 12, 17, or 22 (*P* > 0.05). The variance of weights of both low BW and Av BW pigs increased significantly with each weighing (*P* < 0.001, respectively). Numerically, the greatest increase in variance for the low BW pig population occurred between weeks 12 and 17, whereas for Av BW pigs, the greatest increase in variance occurred between weeks 17 and 22 (Table 3). The majority of low BW pigs ranged from 6 to 8 kg at weaning (54%), whereas Av BW pigs predominantly ranged from 7 to 10 kg (57%). At slaughter age, the majority of low BW pigs ranged from 85 to 105 kg (63%). The majority of Av BW pigs ranged from 90 to 110 kg (58%) at slaughter age. At slaughter age, 24% of low BW pigs recorded a weight which was equal to or greater than the average weight of Av BW pigs.

**Table 3.** The effect of birth weight on the variance in animal liveweight recorded at various ages for low birthweight and average birthweight pigs

	Variance, kg^2^		*P*-value
	Low BW	Av BW	
4 weeks weight	1.95	3.42	<0.001
8 weeks weight	15.39	20.08	0.04
12 weeks weight	44.49	48.71	0.48
17 weeks weight	109.30	109.10	0.99
22 weeks weight	148.80	178.60	0.16

Low BW, low birthweight pigs, <1 kg; Av BW, average birthweight pigs, 1.3 to 1.7 kg.

To demonstrate the impact of the farm on the growth of low BW and Av BW pigs, an interactive statistical approach was taken (Table 4). From birth to week 4, ADG for Av BW pigs was largely similar across the five farms (*P* > 0.05). However, low BW pigs on Farm 5 performed well, matching the ADG of Av BW pigs on all farms (*P* > 0.05), whereas low BW pigs on Farm 4 performed poorer than those of a similar birthweight on the majority of other farms (*P* < 0.05). From weeks 4 to 8, ADG recorded by Av BW pigs on Farm 2 was poor, falling below that of low BW pigs on all other farms (*P* < 0.05). However, low BW pigs on farm 3 recorded an equivalent ADG to Av BW pigs on many other farms (*P* > 0.05). From weeks 17 to 22, low BW animals on farms 3 and 5 matched the ADG of Av BW pigs on other farms (*P* > 0.05). The above growth rates meant that low BW pigs on farms 3 and 5 recorded a liveweight equivalent to that of Av BW pigs on other farms at certain stages of production. Furthermore, Av BW pigs on farm 2 exhibited a liveweight more akin to low BW pigs on other farms from weeks 8 to 22. However, when comparing all farms, low BW animals were lighter than Av BW animals in the majority of cases (*P* < 0.05). Furthermore, when comparing low BW pigs to Av BW pigs on any given farm throughout production, low BW pigs were almost exclusively significantly lighter (*P* < 0.05). The variance in the weight of low BW pigs was significantly different between farms at weeks 8 and 17 (*P* < 0.05; Table 5). However. there was no significant difference at week 4, 12, or 22 (*P* > 0.05). Variance in weight of Av BW pigs varied significantly between farms at weaning, weeks 17 and 22 (*P* < 0.05), but there was no significant difference at week 8 or 12 (*P* > 0.05).

**Table 4.** Effect of farm, birthweight, and their interaction on the liveweight and ADG of low birthweight and average birthweight pigs from birth to 22 weeks of age

							*P*-value		
	Farm 1	Farm 2	Farm 3	Farm 4	Farm 5	SED	Farm	Birthweight	Farm × Birthweight
Liveweight, kg									
Birth									
Low BW	0.97^c^	0.92^ab^	0.89^a^	0.88^a^	0.93^b^	0.020	0.129	<0.001	0.006
Av BW	1.51^d^	1.52^d^	1.52^d^	1.50^d^	1.52^d^				
Week 4									
Low BW	6.91^bc^	6.68^b^	6.26^ab^	5.73^a^	7.45^cd^	0.410	0.086	<0.001	<0.001
Av BW	7.62^cde^	7.45^cde^	8.04^de^	8.08^de^	8.24^e^				
Week 8									
Low BW	16.3^cd^	11.4^a^	17.8^de^	15.4^c^	17.5^de^	0.84	<0.001	<0.001	<0.001
Av BW	19.1^ef^	13.6^b^	22.8^g^	20.4^f^	19.5^f^				
Week 12									
Low BW	33.7^cd^	25.0^a^	35.1^de^	30.3^b^	33.5^cd^	1.45	<0.001	<0.001	0.024
Av BW	37.8^ef^	31.3^bc^	42.2^g^	38.3^f^	36.9^ef^				
Week 17									
Low BW	64.4^de^	49.7^a^	58.2^b^	55.3^b^	62.7^cd^	1.84	<0.001	<0.001	0.022
Av BW	70.4^f^	59.5^bc^	67.5^ef^	65.8^de^	65.6^de^				
Week 22									
Low BW	93.6^cd^	86.7^ab^	92.0^bc^	85.2^a^	99.1^de^	3.06	<0.001	<0.001	0.032
Av BW	101.9^ef^	94.2^cd^	102.4^ef^	100.7^ef^	103.7^f^				
ADG, g/d									
Birth–week 4									
Low BW	211^bcd^	206^bc^	191^ab^	173^a^	232^cd^	12.2	0.148	<0.001	<0.001
Av BW	218^bcd^	213^bcd^	233^cd^	235^d^	240^d^				
Weeks 4 to 8									
Low BW	334^c^	161^a^	409^d^	339^c^	358^c^	23.2	<0.001	<0.001	0.019
Av BW	408^d^	210^b^	526^e^	438^d^	405^d^				
Weeks 8 to 12									
Low BW	623	488	612	516	576	24.4	<0.001	<0.001	0.077
Av BW	671	625	701	633	623				
Weeks 12 to 17									
Low BW	880	709	651	709	822	28.2	<0.001	<0.001	0.156
Av BW	927	814	723	786	818				
Weeks 17 to 22									
Low BW	845^a^	1012^cde^	951^bc^	845^a^	1047^de^	46.4	<0.001	0.008	0.045
Av BW	888^ab^	965^bcd^	1000^cd^	980^cd^	1093^e^				
Birth to week 22									
Low BW	602^cd^	557^ab^	592^bc^	547^a^	637^de^	16.0	<0.001	<0.001	0.033
Av BW	652^e^	602^cd^	655^e^	644^e^	664^e^				
Weeks 4 to 22									
Low BW	689	635	680	628	727	23.1	<0.001	<0.001	0.068
Av BW	748	685	750	735	759				

Low BW, low birthweight pigs, <1 kg; Av BW = average birthweight pigs, 1.3 to 1.7 kg.

Letters refer to significant differences in weight between Low BW and Av BW pigs on each farm (ie, the interaction between Farm and Birthweight) and this is done separately at each weighing (ie, Birth, Week 4, Week 8, Week 12, Week 17 and Week 22. Differences in letters indicate a difference of *P* < 0.05.

**Table 5.** Homogeneity of variance recorded on each farm for the weight of low and average birthweight pigs from 4 to 22 weeks of age

	*P*-value	
	Low BW	Av BW
4 week weight	0.218	0.047
8 week weight	0.015	0.307
12 week weight	0.199	0.232
17 week weight	0.046	0.017
22 week weight	0.220	0.032

Low BW, low birthweight pigs, <1 kg; Av BW, average birthweight pigs, 1.3 to 1.7 kg.

### Preweaning Mortality

Mortality rate and average age and weight at death are shown in Table 6. The preweaning mortality of low BW pigs was over three times greater than that of Av BW pigs (*P* < 0.001). The preweaning mortalities of low BW pigs also occurred earlier in lactation (*P* < 0.001) with low BW pigs being significantly lighter than Av BW animals at death (*P* < 0.001). The average age of preweaning deaths was also significantly younger for nonfostered pigs compared with fostered animals (8.5 d vs. 16.2 d; *P* = 0.006). Preweaning mortality did not differ significantly between farms (*P* > 0.05), nor was there an interaction between farm and birthweight (*P* > 0.05). The cause of preweaning deaths in low BW and Av BW pigs is shown in Table 7. There was a significant association between piglet birthweight and the cause of preweaning death (*P* = 0.008). Starvation and overlying of piglets were the major causes of preweaning mortalities in low BW pigs, accounting for 49% and 28% of all deaths, respectively. In contrast, 30% of preweaning deaths for Av BW pigs were due to an unknown cause, with a further 22% due to overlying by the sow and 13% due to scouring-related illness.

**Table 6.** Effect of birthweight, sow parity, litter size, and fostering on mortality rate, average age of death, and average weight at death for low birthweight and average birthweight pigs

					*P*-value				
	Low BW	Av BW	SEM	Birthweight	BM parity	BA in litter	SB in litter	Total litter size	Fostered
Preweaning									
Mortality, %	20.9	6.1	—	<0.001	0.875	0.383	0.818	0.304	0.072
Av. day no. at death	9.2	15.4	1.81	<0.001	0.244	0.491	0.566	0.435	0.006
Av. death weight	1.2	2.4	0.23	<0.001	0.425	0.427	0.73	0.485	0.132
Postweaning									
Mortality (%)	10.2	6.8	–	0.185	0.445	0.754	0.358	0.580	0.442
Av. day no. at death	89.9	89.1	10.21	0.936	0.706	0.508	0.924	0.558	0.238
Av. death wt	31.5	31.9	7.12	0.953	0.376	0.837	0.772	0.949	0.251

Low BW, low birthweight pigs, <1 kg; Av BW, average birthweight pigs, 1.3 to 1.7 kg; BM parity, parity of birth mother; BA in litter, number of piglets born alive per litter; SB in litter, number of still born animals per litter.

**Table 7.** Percentage cause of preweaning mortality and body area predisposing postweaning mortality for low and average birthweight pigs

	Low BW	Av BW
Preweaning mortalities		
Hurt by sow	6.2	0.0
Lain on	28.4	21.7
Scouring	3.7	13.0
Splay legged	3.7	8.7
Starvation	49.4	21.7
Weak at birth	0.0	4.3
Unknown	8.6	30.4
Postweaning mortalities		
Alimentary tract	27.7	25.0
Cardiovascular system	5.5	0.0
Muscular/skeletal system	16.6	8.3
Nervous system	5.5	0.0
Respiratory system	16.6	41.6
Systemic infection	11.1	8.3
No. of significant findings	16.6	16.6

Low BW = low birthweight pigs, <1 kg; Av BW = average birthweight pigs, 1.3 to 1.7 kg.

### Postweaning Mortality

Birthweight had no significant impact on the rate of postweaning mortality (*P* > 0.05) (Table 6). There was also no significant difference in the age or weight at death between the two birthweight categories (*P* > 0.05, respectively). The most postweaning deaths recorded within 3 weeks occurred between weeks 5 and 7 or 11 and 13 for low BW pigs (25%, respectively) and between weeks 8 and 10 for Av BW pigs (29.4%). Postweaning mortality differed significantly between farms (*P* < 0.05), but there was no interaction between farm and birthweight (*P* > 0.05). The body systems identified as causing postweaning deaths are qualified in Table 7. There were no significant differences in the causes of postweaning deaths between low BW and Av BW pigs (*P* = 0.935). However, across both birthweights of pigs the main body systems identified as causing postweaning deaths were the respiratory tract (27%) and the alimentary tract (27%).

## DISCUSSION

This study was designed to allow the collection of data that would enable a thorough understanding and quantification of modern commercial performance at an individual pig level. The results generated provide an insight into the commercial impact of the increased prevalence of low birthweight pigs.

### Litter Composition

In the present study, low BW piglets originated from litters with a greater number of piglets born alive on average compared with Av BW piglets. This is in line with findings from Quiniou et al. (2002) and is likely due to IUGR which results in lower birthweight piglets with compromised body structure, metabolism, and physiology (Wang et al., 2017).

### Effect of Birthweight on Growth Performance

The main aim of this study was to compare the performance of low BW and Av BW pigs in a commercial setting. As expected, Av BW pigs outperformed those of low BW throughout their lifetime in terms of weight and ADG. Indeed, the average weight difference of 1.2 kg between low BW and Av BW pigs at weaning further diverged to over 9 kg on average at slaughter age, which is of major commercial significance. This divergence in slaughter weight is greater than that reported by previous studies which showed differences of 7.62 kg (Douglas et al., 2014) and 6.1 kg (Beaulieu et al., 2010) in weight at an equivalent slaughter age between low BW and Av BW pigs. Data from the current study, as noted earlier, represent pigs from larger litters with a greater divergence in birthweight than the work by Douglas et al. (2014) and Beaulieu et al. (2010). This increase confirms an increasing problem associated with compromised animals. When modeling piglet growth, low BW animals were shown to require an additional 11 d to achieve a market weight of 120 kg when compared with Av BW counterparts. This is comparable to the literature where Beaulieu et al. (2010) showed low BW pigs take an additional 10 d to reach market weight. However, when calculated based on ADG from birth to slaughter recorded for low BW and Av BW pigs in this study, low BW animals would require an additional 17 d to reach a slaughter weight of 120 kg. This would be expected as the divergence in birthweight from the current study is greater than that employed by Beaulieu et al. (2010). This finding, combined with only 78% of all trial animals fitting the exponential relationship generated by the model, suggests that a modeling approach is better suited to studies conducted under strict experimental conditions such as those given by Lopez-Verge et al. (2018) and Revilla et al. (2019).

A diverse range of factors contribute to the inferior growth associated with low BW pigs. Larger litters have placed increased demand on sows for milk production. Whilst modern sows may express an improved milk yield, this increase is not sufficient to facilitate the maximal growth of increased litter sizes (De Bettio et al., 2016). Furthermore, the greater dominance value of heavier littermates often results in compromised pigs losing teat disputes and missing an increased number of nursing episodes, which may have contributed to the lower weaning weights of low BW pigs in this study (Le Dividich et al., 2017). However, the reduced growth of low BW pigs was evident throughout the trial period until slaughter. There is a clear consensus in the literature that an inferior muscle fiber composition and development is central to the impaired performance of low BW pigs. low BW animals have been shown to possess 19% fewer muscle fibers on average than heavier animals at birth (Gondret et al., 2005), with the difference in fiber number of 85,000 at birth increasing to 250,000 by slaughter (Rehfeldt and Kuhn, 2006). This restricts lean growth capacity and hence weight gain in low BW animals.

Many previous studies have concluded that pig weaning weight is a critical factor in determining lifetime performance, with pigs exhibiting lower weaning weights recording slower growth and higher mortality throughout the rearing and finishing phases (Fix et al., 2010; Williams, 2003). This premise is supported by the findings from the current study where both birthweight and weaning weight were strongly correlated to future performance.

Interestingly, in the current study, the difference in ADG of low BW and Av BW pigs was most pronounced from weeks 4 to 8 and 8 to 12. It is widely recognized that the majority of pigs experience a period of suboptimal growth following weaning, commonly referred to as a postweaning growth check (Tokach et al., 2003). Findings from this study suggest that this growth check is more pronounced in low BW pigs. It is possible that impaired digestive development, which is common in low BW animals, reduced the ability of their gastrointestinal tract to achieve the rapid changes in size, protein turnover, and microbiota composition required at weaning (Pluske et al., 2018). This highlights the immediate postweaning period as a critical window for intervention in low BW pig performance.

### Effect of Fostering on Growth

Animals that had been cross-fostered in this study, particularly those of Av BW, exhibited a lighter weight at multiple stages of the trial period. There is evidence in the literature to suggest that cross-fostering can negatively impact growth (Dewey et al., 2008). For example, Giroux et al. (2000) found fostered piglets to weigh 24% less than nonfostered animals at weaning. This lighter weaning weight has been linked to aggressive fighting between adopted and resident piglets (Wattanaphansak et al., 2002). However, Calderon-Diaz et al. (2018) showed fostering to have no detrimental impact on weaning weight. Producers must therefore ensure sufficient time has been afforded to allow colostrum uptake from the animal’s birth mother before fostering (Rutherford et al., 2013), and avoid repeated fostering where possible (Robert et al., 2001), to minimize any negative effects associated with this increasingly essential management practice. It is worthy to note that the majority of fostered animals were of low BW (57%), which may have influenced these findings.

### Impact of Birthweight and Farm on Growth Variation

A secondary aim of the current study was to compare growth variation between farms. The greater variance in weight of the Av BW pig population in the immediate postweaning period was unexpected, especially as they recorded a superior ADG during this time. Indeed, Milligan et al. (2001) reported a tendency for low BW pigs to record a greater coefficient of variation in weaning weight than Av BW pigs (0.18 vs. 0.15, *P* = 0.081). Results from the present study would suggest that some Av BW pigs experienced elevated growth compared with others immediately postweaning, resulting in a greater variation than that seen in the low BW pig population. Logically, the variance of weight within both low BW and Av BW pig populations increased with age (Pardo et al., 2013). However, Schinckel et al. (2010) quantified the impact of birthweight on growth performance and showed a decreasing influence in the growing and finishing phase. Indeed, birthweight accounted for 13% of the variability in ADG at a liveweight of 46.7 kg, yet only 2% of the variability in ADG at a liveweight of 102 kg. Environmental conditions and genetic differences have been cited as other major factors influencing this variation (Magowan et al., 2007).

Interestingly, the spread of weights at slaughter age was similar for both birthweight categories. Indeed, the spread of weights recorded at slaughter age was numerically greater for Av BW pigs compared with those of low BW. This is in contrast to the literature which often concludes that it is the lightweight animals that should be targeted to reduce growth variation (Schinckel et al., 2010). However, as discussed, the majority of previous work has been conducted on controlled research farms where responses are not always reflective of that in the field (Magowan et al., 2007). As weight variation during the growing and finishing stages has a major impact on economic returns, more uniform growth across all birthweights is required to maximize farm efficiency.

When comparing performance between farms, there were significant differences in liveweight, growth rate, and the variance in weight of pigs belonging to both birthweight categories at various stages of production. Generally, low BW pigs were lighter than Av BW pigs when comparing all farms. The inferior weight of Av BW pigs on farm 2 can be explained by a disease challenge faced by this farm during the trial period. However, even when farm 2 is not considered, there were certain instances where the liveweight of low BW pigs on one farm were equivalent to that of Av BW pigs on another farm. Variation in growth between farms is often attributed to differences in genetics, environmental health and management practices (He et al., 2016). Indeed, genetic lines are often selected on their ability to produce larger, faster-growing progeny (Ladinig et al., 2014). However, this alone is unlikely to account for the extent of the variation recorded between farms in this trial, with three of the five farms studied employing genetics from the same breed and still recording significant differences in performance. It is, therefore, more likely that the variation observed in performance between farms was a consequence of environmental health and farm management. For example, a greater variance in weight on some farms may have been promoted by a difference in the number of piglets suckling each sow after cross-fostering, as this increases the competition for productive teats. Creep feeding is often supplied to piglets during lactation to reduce the load on lactating sows. However, multiple studies have shown creep feeding does not necessarily improve pre- and postweaning growth or litter uniformity, as not all piglets consume the creep offered (Sulabo et al., 2010; Muns and Magowan, 2018). This is in agreement with the current study as low BW animals on farm 5 recorded the greatest weight at weaning and slaughter, despite this being the only farm to not offer creep feed during lactation. Additionally, work by Magowan et al. (2007) comparing growth variation between commercial herds also compared the performance of a subsample of animals from the different herds when reared in a controlled common environment. Results showed the top and bottom performing herds in the common environment differed to the top and bottom performing herds “on farm,” highlighting the impact of management and environmental conditions on performance.

### Effect of Birthweight and Fostering on Preweaning Mortality

The significantly greater preweaning mortality of low BW pigs compared with heavier counterparts agrees with the findings in the literature (Yuan et al., 2015). Furthermore, the reduced age and weight of low BW pigs at death, combined with the clear association between birthweight and cause of preweaning death, shows how mortality manifests differently within different birthweight categories. In the current study, almost half of the preweaning deaths among low BW pigs were due to starvation. This is likely to be linked to the reduced vitality of these piglets at birth. Indeed, as discussed earlier, heavier littermates have been shown to record a significantly greater dominance value, win the majority of teat disputes and gain access to more productive anterior teats (Le Dividich et al., 2017). This restricts the availability and intake of colostrum and milk in compromised animals during early lactation. This can help explain their high level of death due to starvation, as well as earlier preweaning deaths. The high surface area: bodyweight ratio of low BW piglets, combined with a low body fat reserve, increases susceptibility to postnatal hypothermia (Yuan et al., 2015). This can increase the likelihood of crushing due to the lethargic movement of chilled piglets, explaining the high number of low BW pigs that died following overlying by the sow during lactation in the current trial.

Preweaning deaths of average birthweight pigs were less frequent. As these deaths occurred significantly later during lactation compared with lighter contemporaries, it would suggest that these heavier pigs were not affected to the same extent by impaired vitality and milk acquisition in the immediate postnatal period. However, with a large proportion of deaths being attributed to overlying (22%) and scouring-related illness (13%), there is still room for improvement within the heavier pig population.

Findings from the study are in line with the literature, where preweaning mortality tended to be greater in fostered pigs (Calderón Díaz et al., 2018). As discussed previously, fostering can have a negative impact on piglets by inducing stress, restricting colostrum intake, disrupting suckling behavior and can even result in rejection by the foster mother (Dewey et al., 2008; Rutherford et al., 2013). However, it is acknowledged that the majority of fostered animals in this study were low BW pigs and this may have biased the data and results.

### Effect of Birthweight on Postweaning Mortality

There was no significant association between birthweight and cause, weight or age of postweaning death. It was interesting to note that both low BW and Av BW populations recorded a highly similar average day of postweaning death. Furthermore, postweaning mortalities were evenly spread over a wide range of weeks for both birthweight categories, meaning it was not possible to establish a period of “highest mortality risk.” Whilst low BW animals recorded 3.3% greater mortality than those of Av BW during the postweaning period, this difference was not significant. This is in contrast to previous studies showing lightweight pigs to record significantly lower survival rates following weaning (Collins et al., 2017). It was interesting to note that the postweaning mortality rate of Av BW pigs was greater than their rate of mortality preweaning. This is an area of concern for producers as in addition to reducing income through a reduced number of pigs marketed, postweaning deaths also represent a wasted investment in terms of feed costs.

The alimentary tract was found to be affected in 27.7% of low BW and 25% of Av BW postmortem examinations. This concurs with Edwards et al. (2013) who demonstrated gastric diseases can account for up to 60% of mortalities during the weaner phase. It is thought the abrupt withdrawal of maternal milk at weaning, which supplies a variety of bioactive compounds to aid digestive and immune development, can contribute to digestive disorders in the postweaning period. Animals can also suffer deleterious changes to intestinal structure and function due to insufficient feed intake postweaning. This can lead to intestinal inflammation which compromises the villus-crypt architecture and gastrointestinal tract barrier function as well as disrupting intestinal microbiota (Moeser et al., 2017).

The respiratory tract was also implicated in 16.6% of low BW and 41.6% of Av BW postweaning mortalities in this trial. High animal stocking densities, inadequate ventilation, and failure to maintain house hygiene can increase the risk of respiratory disease (Maes et al., 2008). A variety of approaches including improved feeding conditions, eradication schemes, and genetic selection for improved host immunity have shown promise (Huang et al., 2017). However, further work is still required in this area.

## CONCLUSIONS

This study has found that within modern pig production in a commercial setting, low BW pigs had a 15% greater preweaning mortality, 56 g/d inferior growth rate and 10% lighter slaughter weight on average compared with Av BW pigs. This confirms low BW pigs are a chronic problem at the farm level. At slaughter age, it was notable that 24% of low BW pigs recorded a weight which was equal to or greater than the average weight of Av BW pigs, indicating some low BW animals exhibit acceptable performance. Findings from this study show that targeted intervention is essential to minimize this birthweight associated performance differential, with the lactation and the immediate postweaning periods highlighted as potential targets. This requires further investigation.

## Supplementary Material

txaa147_suppl_Supplementary_Material
