# Supplementary material for: What is the current significance of low birthweight pigs on commercial farms in Northern Ireland in terms of impaired growth and mortality?
Source: Transl Anim Sci. 2020 Jul 31;4(3):txaa147. doi: 10.1093/tas/txaa147 (PMC7485618; doi:10.1093/tas/txaa147)

What is the current significance of low birthweight pigs on commercial farms in Northern Ireland in terms of impaired growth and mortality?

S. J. Hawe*†, N. Scollan†, A. Gordon* and E. Magowan*†

**Agri-Food and Biosciences Institute, Hillsborough, Northern Ireland BT26 6DR*

† *Queens University Belfast, University Road, Belfast, Northern Ireland, BT7 1NN*

Corresponding author: Samuel Hawe ([shawe02@qub.ac.uk](mailto:shawe02@qub.ac.uk))

**Table 1.** Profile of animal usage, genetics and management for each farm

|  | Farm 1 | Farm 2 | Farm 3 | Farm 4 | Farm 5 |
| --- | --- | --- | --- | --- | --- |
| **Gestation accommodation** |  |  |  |  |  |
| Gestation pen type | Open space | Voluntary cubicles | Open space | Voluntary cubicles | Voluntary cubicles |
| Gestation pen group size | 8 | 22 | 20 | 3 - 14 | 4 |
|  |  |  |  |  |  |
| **Farrowing accommodation** |  |  |  |  |  |
| No. Piglets used | 113 (59 Low BW, 54 Av BW) | 108 (54 Low BW, 54 Av BW) | 119 (64 Low BW, 55 Av BW) | 120 (62 Low BW, 58 Av BW) | 161 (89 Low BW, 71 Av BW) |
| No. boars (B) / gilts (G) | Low BW = 31B 28G Av BW = 28B 26G | Low BW = 28B 26G Av BW = 27B 27G | Low BW = 31B 33G Av BW = 28B 27G | Low BW = 30B 32G Av BW = 30B 28 G | Low BW = 43B 46G Av BW = 37B 34G |
| Parity of origin | Low BW = 3.52, Av BW = 3.70 | Low BW = 2.21, Av BW = 2.19 | Low BW = 2.44, Av BW = 2.38 | Low BW = 3.76, Av BW = 3.60 | Low BW = 4.08, Av BW = 4.21 |
| Piglet genotype | (Topigs Z-line semen x  (Large White x Landrace sows)) | (Duroc Semen x  (Large White x Landrace sows)) | (Duroc Semen x  (Large White x Landrace sows)) | (PIC 337 Semen x  (Large White x Landrace sows)) | (Duroc Semen x  (Large White x Landrace sows)) |
| No. sows used | 19 | 30 | 19 | 18 | 57 |
| Range of sow parities | 1 - 7 | 1-6 | 1-6 | 1 - 7 | 1 - 7 |
| Pen dimension, m^2^ | 4.86 | 4 | 4.32 | 4.86 | 3.45 |
| Farrowing induced | Yes | Yes | Yes | No | Yes |
| Heat pads used | Yes | Yes | Yes | Yes | No |
| Enclosed heated creep area | No | No | No | No | Yes |
| Heat lamps used | Yes | No | No | No | Yes |
| Supplementary milk employed | No | No | Yes | No | No |
| Temperature, ^o^C | 20 - 22 | 21-24 | 21 - 23 | 22.5 | 17.5 - 19 |
| Time of teeth clipping and tail docking | Within 24 hours | Within 24 hours | Within 24 hours | Within 24 hours | Within 24 hours |
| Creep feed offered | Yes | Yes | Yes | Yes | No |
| Cross fostering policy | Piglets fostered 12 hours post-farrowing based on litter size to ensure even litters of approx. 14 pigs | Smallest piglets fostered onto gilts within 24 hours of birth to give even litter sizes | Smallest piglets fostered onto parity 2/3 sows 24 hours post-farrowing | Piglets fostered 24 hours post-farrowing based on litter size to ensure litters of approx. 15 pigs | Piglets fostered 36 hours post-farrowing based on litter size to ensure litters of 15 pigs |
| Conditions vaccinated against | PRRS, PCV2, *Mycoplasma hypopneumoniae* | PCV2, *Mycoplasma hypopneumoniae* | PRRS, PCV2, *Mycoplasma hypopneumoniae* | PRRS, PCV2, *Mycoplasma hypopneumoniae* | PCV2, *Mycoplasma hypopneumoniae* |
| Age at vaccination, days | 21 | 30 | 21 | 14 & 28 | 28 |
| Age at weaning, days | 28 | 30 | 27 | 28 | 28 |
|  |  |  |  |  |  |
| **First stage accommodation** |  |  |  |  |  |
| Pen dimension, m^2^ | 5.1 | 20.5 | 14.4 | 7.75 | 3.8 |
| Pigs per pen | 24 | 100 | 45 | 25 | 10 |
| Feeder type | Dry multi-space | Dry multi-space | Wet and dry | Dry multi-space | Dry multi-space |
| Temperature, ^o^C | 22-26 | 24-27 | 22-28 | 23 - 28.5 | 22 - 28 |
| Age at transfer to second stage, days | 56 | 65 | 61 | 42 | (1st and 2nd stage  combined) |
|  |  |  |  |  |  |
| **Second stage accommodation** |  |  |  |  |  |
| Pen dimension, m^2^ | 10.2 | 45 | 18.45 | 14.9 | 3.8 |
| Pigs per pen | 25 | 100 | 45 | 25 | 10 |
| Feeder type | Dry multi-space | Wet long troughs | Wet and dry | Wet and dry | Dry multi-space |
| Temperature, ^o^C | 22-24 | 20-22 | 24 | 23 | 22 - 28 |
| Age at transfer to finish | 84 | 95 | 101 | 77 | 70 |
|  |  |  |  |  |  |
| **Finishing accommodation** |  |  |  |  |  |
| Pen dimension, m^2^ | 22.75 | 18.75 | 45.6 | 25.3 | 14.4 |
| Pigs per pen | 30 | 25 | 60 | 45 | 16 |
| Feeder type | Wet long troughs | Wet and dry | Wet and dry | Wet and dry | Wet and dry |
| Temperature, ^o^C | 20-22 | 21 | 22 | 22 | 20 |
| Age when first in batch slaughtered, days | 155 | 151 | 150 | 160 | 153 |
|  |  |  |  |  |  |

**Table 2.** Dietary regimes offered on each farm

|  | Farm 1 | Farm 2 | Farm 3 | Farm 4 | Farm 5 |
| --- | --- | --- | --- | --- | --- |
| **Gestation diet** | 16% CP^1^, 0.7% Lys^2^ | 13% CP^1^, 0.7% Lys^2^ | 14.2% CP^1^, 0.65% Lys^2^ | 11.93% CP^1^, 0.58% Lys^2^ | 13.6% CP^1^, 0.6% Lys^2^ |
| **Lactation diet** | 17% CP^1^, 1.0% Lys^2^ | 20% CP^1^, 1.3% Lys^2^ | 17% CP^1^, 1.0% Lys^2^ | 19.08% CP^1^, 1.27% Lys^2^ | 17.4% CP^1^, 1.2% Lys^2^ |
| **First stage accommodation** |  |  |  |  |  |
| Diet 1 |  |  |  |  |  |
| *Diet composition* | 17.5% CP^1^, 1.6% Lys^2^ | 22.5% CP^1^, 1.70% Lys^2^ | 17.5% CP^1^, 1.57% Lys^2^ | 20.75% CP^1^, 1.75% Lys^2^ | 22.5% CP^1^, 1.70% Lys^2^ |
| *Time offered* | Day 28-41 | Day 30-34 | Day 27-30 | Day 28-30 | Day 28-33 |
| Diet 2 |  |  |  |  |  |
| *Diet composition* | 20.2% CP^1^, 1.5% Lys^2^ | 20% CP^1^, 1.45% Lys^2^ | 22.5% CP^1^, 1.70% Lys^2^ | 22% CP^1^, 1.55% Lys^2^ | 22% CP^1^, 1.55% Lys^2^ |
| *Time offered* | Day 42-55 | Day 35-64 | Day 31-35 | Day 31-34 | Day 34-41 |
| Diet 3 |  |  |  |  |  |
| *Diet composition* | - | - | 22% CP^1^, 1.55% Lys^2^ | 20% CP^1^, 1.4% Lys^2^ | - |
| *Time offered* | - | - | Day 36-42 | Day 35-41 | - |
| Diet 4 |  |  |  |  |  |
| *Diet composition* | - | - | 20.2% CP^1^, 1.5% Lys^2^ | - | - |
| *Time offered* | - | - | Day 43-60 | - | - |
| **Second stage accommodation** |  |  |  |  |  |
| Diet 1 |  |  |  |  |  |
| *Diet composition* | 17.5% CP^1^, 1.2% Lys^2^ | 20% CP^1^, 1.25% Lys^2^ | 20.5% CP^1^, 1.5% Lys^2^ | 19% CP^1^, 1.3% Lys^2^ | 18.5% CP^1^, 1.4% Lys^2^ |
| *Time offered* | Day 56-84 | Day 65-94 | Day 61-74 | Day 42 - 76 | Day 42-69 |
| Diet 2 |  |  |  |  |  |
| *Diet composition* | - | - | 17.5% CP^1^, 1.2% Lys^2^ | - |  |
| *Time offered* | - | - | Day 75-100 | - |  |

| **Finishing stage accommodation** |  |  |  |  |  |
| --- | --- | --- | --- | --- | --- |
| Diet 1 |  |  |  |  |  |
| *Diet composition* | 15.5% CP^1^, 1.1% Lys^2^ | 17.5% CP^1^, 1.1% Lys^2^ | 16% CP^1^, 1.50% Lys^2^ | 17% CP^1^, 1.1% Lys^2^ | 18.5% CP^1^, 1.4% Lys^2^ |
| *Time offered* | Day 85-slaughter | Day 95-slaughter | Day 101-142 | Day 77 - 100 | Day 70-84 |
| Diet 2 |  |  |  |  |  |
| *Diet composition* | - | - | 15.5% CP^1^, 1.1% Lys^2^ | 16% CP^1^, 1.0% Lys^2^ | 16.7% CP^1^, 1.2% Lys^2^ |
| *Time offered* | - | - | Day 143-slaughter | Day 101-slaughter | Day 85-slaughter |

^1^CP = Crude protein

^2^Lys = Lysine

**Appendix 1.** Template used by all farmers to document pre-weaning mortalities


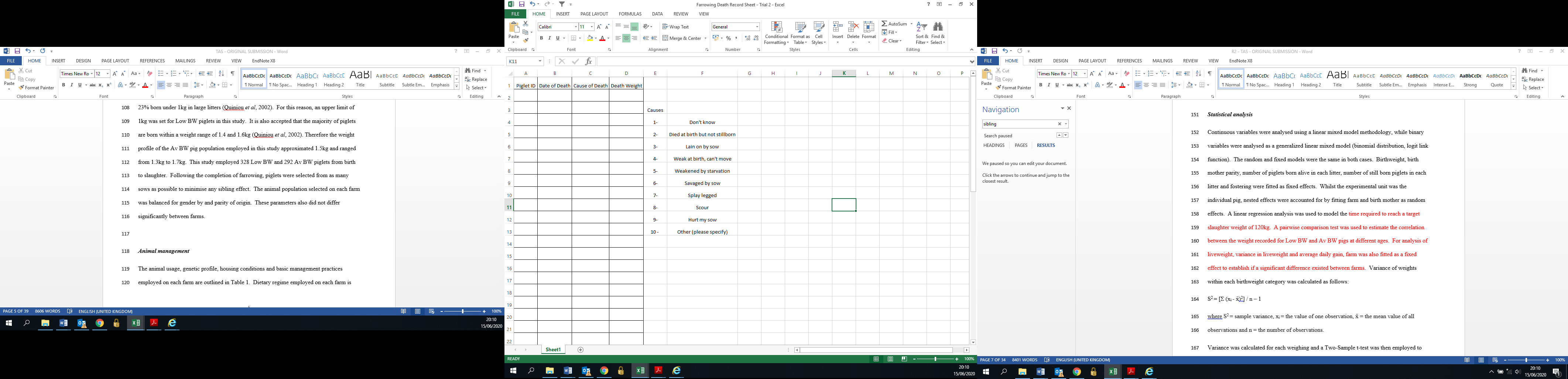

Supplement: txaa147_suppl_Supplementary_Material [file txaa147_suppl_supplementary_material.docx]
